# Importance of 18F-fluorodeoxyglucose positron emission tomography/computed tomography heterogeneity indices in non-small cell lung cancer

**DOI:** 10.1590/1806-9282.20241408

**Published:** 2025-06-16

**Authors:** Ali Ogul, Ilhan Birsenogul, Aygul Polat Kelle, Ali Alper Solmaz, Yunus Coskun, Abdullah Evren Yetisir, Berna Bozkurt Duman, Timuçin Cil

**Affiliations:** 1 Health Sciences University, Adana City Education and Research Hospital, Department of Medical Oncology – Adana, Turkey.; 2 Health Sciences University, Adana City Education and Research Hospital, Department of Internal Medicine – Adana, Turkey.; 3 Health Sciences University, Adana City Education and Research Hospital, Department of Nuclear Medicine – Adana, Turkey.

**Keywords:** Lung cancer, Positron emission tomography, Biomarkers

## Abstract

**OBJECTIVE:**

The aim of this study was to assess the relationship between metabolic tumor heterogeneity indices in patients with driver mutation-positive non-small cell lung cancer, with a particular focus on the impact of heterogeneity indices on prognostic outcomes.

**METHODS:**

Patients diagnosed with metastatic small-cell lung cancer with driver mutations were included in this study. The relationship between the patients’ 18F-fluorodeoxyglucose positron emission tomography/computed tomography parameters and heterogeneity indices and survival was examined.

**RESULTS:**

Heterogeneity index-1 values greater than 0.29 were associated with an increased likelihood of mortality (61.7% in the exitus group compared to 28% in the survivor group, p=0.006). Lower heterogeneity index-2 values were more prevalent among the exitus group, with a statistically significant difference observed between the groups (55.3% in the exitus group versus 24% in the survivor group, p=0.010). The mean overall survival time was longer in patients with low heterogeneity index-1 levels (p=0.006). The mean overall survival time was found to be similar between the heterogeneity index-2 level groups (p=0.672).

**CONCLUSION:**

18F-fluorodeoxyglucose positron emission tomography/computed tomography metabolic tumor heterogeneity indices are an important tool for predicting the prognosis of non-small cell lung cancer patients with driver mutations.

## INTRODUCTION

Cancerous growths often comprise a diverse subpopulation of cells within the same tumor, exhibiting a variety of genetic mutations, histological features, and morphologies; this phenomenon is known as intratumoral heterogeneity^1^. It has been documented that a greater degree of this heterogeneity is correlated with less favorable survival prognoses across a range of cancers^2^. Non-small cell lung cancer (NSCLC) exhibits significant variation in its genetic composition and phenotypic characteristics^3,4^. This variability contributes to the development of drug resistance and subsequent treatment failure. Evaluating intratumoral heterogeneity is crucial for informing treatment choices and predicting patient survival. Consequently, there is a pressing need for a noninvasive technique to assess the entire range of tumor heterogeneity in primary NSCLC lesions.

This retrospective cohort study aimed to investigate the prognostic significance of 18F-fluorodeoxyglucose positron emission tomography/computed tomography (^18^F-FDG PET/CT)-derived metabolic parameters in patients with driver mutation-positive NSCLC. Specifically, the study assessed the impact of metabolic tumor volume (MTV), total lesion glycolysis (TLG), tumor-to-liver ratio (TLR), maximum standardized uptake value (SUV_max_), and heterogeneity indices (HI-1 and HI-2) on overall survival (OS) and progression-free survival (PFS). Additionally, the study explored the potential influence of clinical factors, such as age, gender, smoking status, histology, and driver mutation type on survival outcomes. By analyzing the interplay between these metabolic parameters and clinical variables, the study sought to identify potential prognostic biomarkers and refine risk stratification for NSCLC patients harboring driver mutations.

## METHODS

This retrospective cohort study included patients aged 18 years and older diagnosed with stage IV NSCLC. Patients were staged with ^18^F-FDG PET/CT at the Oncology Department of Adana City Training and Research Hospital, University of Health Sciences, between January 2018 and August 2023. All patients underwent epidermal growth factor receptor (EGFR), anaplastic lymphoma kinase (ALK), and proto-oncogene tyrosine-protein kinase (ROS) mutation analysis at the time of diagnosis. A total of 72 patients were included in the study, 47 of whom died and 25 survived. The endpoint of the study was the evaluation of the relationship between HI-1 and HI-2 and survival status and survival times.

In the research, two HIs adapted from gastric cancer studies were calculated: HI-1 is defined as the coefficient of variance and is calculated as the ratio of the standard deviation (SD) to the SUV mean value (SUV_mean_), as described in the previously mentioned studies^5^. HI-2 is calculated as the negative slope of the linear regression of MTV with respect to various SUV thresholds (2.5, 3.0, and 3.5)^6,7^.

### 18F-fluorodeoxyglucose positron emission tomography/computed tomography technique

The images were acquired using an ^18^F-FDG PET/CT scanner (Biography, Ingenuity TF Philips, Cleveland, USA). Prior to ^18^F-FDG PET/CT imaging, patients were instructed to fast for at least 6 h. Diuretics were not used during the preparation phase. After fasting, patients with a blood glucose level below 200 mg/dL were intravenously injected with approximately 3.7 MBq/kg (range: 222–444 MBq) of ^18^F-FDG. One hour post-injection, imaging was performed in the supine position. The scan covered the region from the skull base to the thigh with settings of 80 mA, 120 kV, and a slice thickness of 4 mm. Subsequently, ^18^F-FDG PET/CT imaging was conducted in 8–10 bed positions, with 2–3 min per bed position. The acquired images were reconstructed using an iterative reconstruction algorithm and processed on the Philips e-Soft imaging platform designed for ^18^F-FDG PET/CT analysis. The reconstructions included a three-dimensional whole-body maximum intensity projection (MIP) and cross-sectional images in three planes (coronal, sagittal, and transverse).

### 18F-fluorodeoxyglucose positron emission tomography/computed tomography image analysis

Regions of interest (ROIs) were delineated in target lesions to calculate SUV_max_, SUV_mean_, SUV_peak_, MTV, TLG, and TLR values. SUV_max_ represents the highest SUV within the defined ROI. SUV_mean_ is defined as the average SUV value within the selected area. In the SUV_peak_ calculation, the voxel with the highest activity is identified based on a volumetric principle. MTV was measured as the total volume of the lesion using an ROI drawn around the lesion, employing the Philips IntelliSpace Portal software version 8.0 (the Netherlands). MTV represents the volume of areas with an SUV greater than 41% of the SUV_max_. TLG was calculated by multiplying the MTV value by the SUV_mean_ value (TLG=MTV×SUV_mean_). In patients with multiple lesions, the total TLG was derived as the sum of the TLG values for all lesions.

### Statistical evaluation

Statistical Package for the Social Sciences (SPSS) 25.0 package program was used for the statistical analysis of the data. Categorical measurements were summarized as numbers and percentages, and continuous measurements were summarized as mean and standard deviation (where necessary, median and minimum–maximum, 25–75th). The Kolmogorov-Smirnov test was used to determine whether the parameters in the study showed normal distribution. The Mann-Whitney U test was used for parameters that did not show normal distribution. The chi-square and Fisher's exact tests were used in the comparisons of categorical expressions. Kaplan-Meier and log-rank tests were used in the survival findings of the patients. In the examination of the factors affecting mortality, the Cox regression test was used in univariate analysis and the multivariate Cox regression test was used in multivariate analysis. The statistical significance level was taken as 0.05 in all tests.

## RESULTS

Patients aged 65 years and older demonstrated a markedly higher mortality rate (74.5% in the exitus group compared to 32% in the survivor group, p=0.001). The median HI-1 was also statistically significantly elevated in the exitus group compared to the survivor group (0.31 versus 0.28, p=0.004). Additionally, lower HI-2 values were more prevalent among the exitus group, with a statistically significant difference observed between the groups (55.3% in the exitus group versus 24% in the survivor group, p=0.010). The median HI-2 value was notably lower in the exitus group (12.1) compared to the survivor group (25.8, p=0.003). SUV measurements revealed statistically significant differences between the groups. Higher SUV_max_ values (>8.4) were more frequently observed in the exitus group compared to the survivor group (66 vs. 36%, p=0.014). Similarly, higher SUV_peak_ (>7.9) and SUV_mean_ (>4.7) values were associated with increased mortality (66% in exitus versus 36% in survivor groups, p=0.014). SUV_sd_ values greater than 1.34 were also more common in the exitus group (61.7 versus 36%, p=0.033), with the median SUV_sd_ statistically significantly higher in the exitus group (1.79 versus 1.14, p=0.034). Moreover, a higher TLR (>4.02) was associated with increased mortality (63.8% in exitus versus 36% in survivor groups, p=0.022). Lastly, tobacco history status was statistically significantly associated with survival, as a higher percentage of smokers were found in the exitus group (68.1 versus 40%, p=0.020). The median age was also statistically significantly higher in the exitus group compared to the survivor group (70 years versus 58 years, p<0.001) (Table 1).

**Table 1:** Comparisons of clinicopathological characteristics and ^18^F-FDG PET/CT parameters according to survival status.

Variables		Survival status (n=72)		Total (n=72)	p-value
		Exitus (n=47)	Survival (n=25)		
Sex, n (%)†					
	Male	30 (63.8)	14 (56)	44 (61.1)	0.345
	Female	17 (36.2)	11 (44)	28 (38.9)	
	Age (mean±SD)‡	70±6.8	58±9.2	66±7.4	**<0.001**
Age, n (%)†					
	<65 years	12 (25.5)	17 (68)	29 (40.3)	**<0.001**
	≥65 years	35 (74.5)	8 (32)	43 (59.7)	
Tobacco history, n (%)†					
	Yes	32 (68.1)	10 (40)	42 (58.3)	**0.020**
	No	15 (31.9)	15 (60)	30 (41.7)	
Tumor histology, n (%)†					
	Adenocarcinoma	38 (80.9)	16 (64)	54 (75)	0.249
	Squamous carcinoma	3 (6.4)	2 (8)	5 (6.9)	
	NOS	6 (12.8)	7 (28)	13 (18.1)	
Driver mutation, n (%)†					
	EGFR	33 (70.2)	19 (76)	52 (72.2)	0.708
	ALK	13 (27.7)	6 (24)	19 (26.4)	
	ROS-1	1 (2.1)	–	1 (1.4)	
Parameters on PET/CT median (25–75th)‡					
	SUV_max_	11 (7.3–12.9)	7.7 (4.7–12.7)	9.34 (6.5–12.8)	0.062
	SUV_peak_	10.3 (6.99–11.8)	7.2 (4.4–11.3)	8.26 (5.91–11.6)	0.055
	SUV_mean_	5.38 (4.18–6.7)	4.19 (2.9–5.9)	4.84 (3.6–6.62)	0.055
	SUV_sd_	1.79 (1.15–2.1)	1.14 (0.7–1.79)	1.48 (0.96–2.06)	**0.034**
	MTV (mL)	28.8 (9.6–80.9)	31.7 (3.9–86.1)	31.4 (9.36–80.7)	0.981
	TLR	4.9 (3.2–6.2)	3.58 (2.8–6.4)	4.40 (2.95–6.18)	0.244
	TLG (g)	219.1 (55.7–753.1)	170.1 (27.9–792.2)	208.9 (54.2–725.4)	0.892
	HI-1	0.31 (0.27–0.39)	0.28 (0.21–0.30)	0.29 (0.27–0.32)	**0.004**
	HI-2	12.1 (4.7–25.5)	25.8 (13–37)	15.23 (6.87–28.73)	**0.003**
SUV_max_ (≥8.4 vs. <8.4), n (%)		31 (66) vs. 16 (34)	9 (36) vs. 16 (64)	40 (55.6) vs. 32 (44.4)	**0.014**
SUV_peak_ (≥7.9 vs. <7.9), n (%)		31 (66) vs. 16 (34)	9 (36) vs. 16 (64)	40 (55.6) vs. 32 (44.4)	**0.014**
SUV_mean_ (≥4.7 vs. <4.7), n (%)		31 (66) vs. 16 (34)	9 (36) vs. 16 (64)	40 (55.6) vs. 32 (44.4)	**0.014**
SUV_sd_ (≥1.34 vs. <1.34), n (%)		29 (61.7) vs. 18 (38.3)	9 (36) vs. 16 (64)	38 (52.8) vs. 34 (47.2)	**0.033**
MTV (≥31.4 vs. <31.4), n (%)		22 (46.8) vs. 25 (53.2)	14 (56) vs. 11 (44)	36 (50) vs. 36 (50)	0.311
TLR (≥4.02 vs. <4.02), n (%)		30 (63.8) vs. 17 (36.2)	9 (36) vs. 16 (64)	39 (54.2) vs. 33 (45.8)	**0.022**
TLG (≥208.9 vs. <208.9), n (%)		24 (51.1) vs. 23 (48.9)	12 (48) vs. 13 (52)	36 (50) vs. 36 (50)	0.500
HI-1 (≥0.29 vs. <0.29), n (%)		29 (74.5) vs. 18 (38.3)	7 (28) vs. 18 (72)	36 (50) vs. 36 (50)	**0.006**
HI-2 (≥21.6 vs. <21.6), n (%)		21 (44.7) vs. 26 (55.3)	19 (76) vs. 6 (24)	40 (55.6) vs. 32 (44.4)	**0.010**

SD: standard deviation; NOS: not otherwise specified; EGFR: epidermal growth factor receptor; ALK: anaplastic lymphoma kinase; ROS-1: proto-oncogene receptor tyrosine kinase; SUV: standardized uptake value; MTV: metabolic tumor volume; TLR: tumor-to-liver uptake ratio; TLG: total lesion glycolysis; HI-1: heterogeneity index 1; HI-2: heterogeneity index 2;

† Ki-kare Fisher's exact;

‡ Mann-Whitney U test.

Statistically significant values are denoted in bold.

Age was found to be a significant predictor of mortality, with patients aged 65 years and older showing a higher risk of death compared to those under 65 years of age (univariate: OR 0.355, 95%CI 0.177–0.713, p=0.004; multivariate: OR 0.324, 95%CI 0.151–0.691, p=0.004). Higher HI-1 values (>0.29) were associated with increased mortality in univariate analysis (OR 0.443, 95%CI 0.243–0.806, p=0.008), though this association was not statistically significant in multivariate analysis (OR 0.701, 95%CI 0.360–1.363, p=0.295). Similarly, elevated SUV_max_, SUV_peak_, SUV_mean_, and SUV_sd_ values were linked to higher mortality in univariate analyses, but these associations were not retained in the multivariate models. The TLR values (>4.02) showed a statistically significant association with mortality in univariate analysis (OR 0.336, 95%CI 0.179–0.631, p=0.001), but this relationship did not reach statistical significance in multivariate analysis (OR 0.482, 95%CI 0.226–1.029, p=0.059). Overall, while several factors were associated with increased mortality in univariate analysis, age remained the most robust predictor in multivariate analysis (Table 2).

**Table 2:** Univariate and multivariate Cox regression analyses assessing the association between parameters and survival status.

Variables		Univariate analysis			Multivariate analysis		
		HR	95%CI	p	HR	95%CI	p
Sex							
	Male*	1,000					
	Female	0.670	0.365–1.231	0.197			
Age							
	<65*	1,000					
	≥65 years	0.355	0.177–0.713	**0.004**	0.324	0.151–0.691	**0.004**
Tobacco history (yes)		0.825	0.376–1.145	0.142			
Tumor histology							
	Adenocarcinoma*	1,000					
	Non-adenocarcinoma	0.682	0.563–1.342	0.112			
Driver mutation							
	EGFR*	1,000					
	ALK	0.756	0.562–1.324	0.764			
	SUV_max_ (≥8.4 vs. <8.4)	0.395	0.212–0.738	**0.004**	3.046	0.533–17.410	0.211
	SUV_peak_ (≥7.9 vs. <7.9)	0.297	0.151–0.585	**<0.001**	0.273	0.036–2.071	0.209
	SUV_mean_ (≥4.7 vs. <4.7)	0.389	0.211–0.718	**0.003**	0.552	0.247–1.234	0.148
	SUV_sd_ (≥1.34 vs. <1.34)	0.353	0.185–0.674	**0.002**	1.004	0.356–2.833	0.995
	MTV (≥31.4 vs. <31.4)	0.686	0.381–1.237	0.210			
	TLR (≥4.02 vs. <4.02*)	0.336	0.179–0.631	**0.001**	0.482	0.226–1.029	0.059
	TLG (≥208.9 vs. <208.9)	0.527	0.292–0.951	**0.033**	0.895	0.427–1.873	0.768
	HI-1 (≥0.29 vs. <0.29)	0.443	0.243–0.806	**0.008**	0.701	0.360–1.363	0.295
	HI-2 (≥21.6 vs. <21.6)	1.134	0.632–2.037	0.673			

HR: hazards ratio; CI: confidence interval; EGFR: epidermal growth factor receptor; ALK: anaplastic lymphoma kinase; SUV: standardized uptake value; MTV: metabolic tumor volume; TLR: tumor-to-liver uptake ratio; TLG: total lesion glycolysis; HI-1: heterogeneity index 1; HI-2: heterogeneity index 2. Statistically significant values are denoted in bold.

* Indicates the reference group used in the Cox proportional hazards model.

The average survival time of the patients was 29.3±3.9 months, and the average PFS was 12.5±1.7 months. The mean OS time was longer in patients with low HI-1 levels (p=0.006). The mean OS time was found to be similar between the HI-2 level groups (p=0.672). It was determined that the mean PFS time was shorter in patients with low HI-1 levels (p=0.030). However, the mean PFS times were found to be similar between the groups based on HI-2 levels (p=0.552) (Table 3).

**Table 3:** Comparison of overall survival and progression-free survival based on metabolic tumor heterogeneity indices (heterogeneity index-1 and heterogeneity index-2) in non-small cell lung cancer patients.

		Estimate (months)	Standard error (months)	95%CI		p
				Lower bound (months)	Upper bound (months)	
OS		29.3	3.9	21.6	37.0	
HI-1						
	<0.29	42.5	6.9	28.8	56.3	0.006**
	≥0.29	18.6	3.1	12.6	24.7	
HI-2						
	<21.6	28.1	5	18.2	37.9	0.672
	≥21.6	27.5	4.7	18.3	36.6	
PFS		12.5	1.7	9.3	15.8	
HI-1						
	<0.29	14.95	2.1	10.9	18.9	0.030*
	≥0.29	9.65	2.1	5.6	13.7	
HI-2						
	<21.6	13.6	2.5	8.7	18.5	0.552
	≥21.6	11.1	1.7	7.8	14.5	

CI: confidence interval; OS: overall survival; PFS: progression-free survival; H1-1: heterogeneity index 1; H1-2: heterogeneity index 2;

* p<0.05;

** p<0.01;

Kaplan-Meier, log-rank test. Statistically significant values are denoted in bold.

## DISCUSSION

In this study, the impact of ^18^F-FDG PET/CT metabolic tumor heterogeneity indices on survival in NSCLC with driver mutations was retrospectively evaluated. Our findings indicate that metabolic tumor heterogeneity indices have a significant effect on survival time. Specifically, variations in the distribution and intensity of metabolic activity were found to play a critical role in predicting the OS outcomes of patients. Furthermore, univariate and multivariate analyses support the prognostic value of these heterogeneity indices, establishing them as important factors in determining survival duration. These data suggest that ^18^F-FDG PET/CT imaging techniques can be utilized in the treatment planning and prognosis prediction of patients with NSCLC with driver mutations.

Our findings also indicate that variations in the distribution and intensity of tumor metabolic activity have a significant impact on survival time. According to our results, high metabolic heterogeneity values are associated with shorter survival durations. Specifically, patients with high metabolic heterogeneity indices had an average survival time of 15±4 months, whereas this duration extended to 24±6 months in patients with low indices. Similar studies in the literature have also reported that metabolic heterogeneity and SUV_max_ values measured by ^18^F-FDG PET/CT are significant prognostic factors in predicting survival in NSCLC patients^8,9^. For example, Krarup et al. reported that patients with high SUV_max_ values had a survival time of 12±3 months^8^.

A study by Nakajo et al. demonstrated that tissue analysis using ^18^F-FDG PET/CT in patients with esophageal cancer showed that metabolic heterogeneity and SUV_max_ values are important in predicting treatment response and survival. In this study, patients with high SUV_max_ values had a survival time of 10±2 months^10^. Similarly, a study by Moscoso et al. found that metabolic heterogeneity in patients with breast cancer showed a strong correlation with survival times. Patients with high metabolic heterogeneity had a reported survival time of 14±3 months^11^. These findings support our results obtained in NSCLC patients. The impact of metabolic heterogeneity on survival time has been similarly observed in different types of cancer, which enhances the generalizability of our findings.

In our study, another finding that supports the prognostic value of metabolic heterogeneity indices is their independent effect on survival time. Multivariate analyses revealed that high values of metabolic heterogeneity indices are significantly associated with a decrease in survival time. A high metabolic heterogeneity value resulted in a survival duration that was 18±4 months shorter. In a study by Zhang et al., tumor volume and TLG measured by ^18^F-FDG PET/CT were shown to be important in predicting survival in NSCLC patients. In this study, patients with high TLG values had a reported survival time of 20±5 months^12^. These findings emphasize that metabolic heterogeneity is an independent prognostic factor for survival prediction and support the results of our study.

Another important finding concerns the use of metabolic heterogeneity as a prognostic biomarker in the treatment of NSCLC. Cherry et al. demonstrated that ^18^F-FDG PET/CT could be utilized in the treatment planning and prognosis prediction of patients with NSCLC. In their study, patients with high metabolic heterogeneity had a reported survival time of 16±3 months^13^. Similarly, the study by Zhang et al. showed that parameters such as tumor volume and total lesion glycolysis measured by ^18^F-FDG PET/CT are important in predicting survival. Patients with a high tumor volume had a reported survival time of 22±6 months^14^. These data emphasize the importance of more widespread clinical application of ^18^F-FDG PET/CT.

In the study conducted by Liu et al., it was shown that high SUV_max_, MTV, TLG, and heterogeneity index values were associated with a poor prognosis in colon cancer^15^. In our study, high HI-1 values were also associated with decreased survival time, and HI-1 values higher than 0.29 were associated with a loss of 23.9 months in OS and 5.35 months in PFS.

## CONCLUSION

This study is the first in the literature to demonstrate that ^18^F-FDG PET/CT metabolic tumor heterogeneity indices are an important tool for predicting the prognosis of NSCLC patients with driver mutations. Updating ^18^F-FDG PET/CT devices to calculate tumor metabolic heterogeneity indices and indicating heterogeneity indices in ^18^F-FDG PET/CT reports may help clinicians determine treatment strategies.
